# Skin Cornification Proteins Provide Global Link between ROS Detoxification and Cell Migration during Wound Healing

**DOI:** 10.1371/journal.pone.0011957

**Published:** 2010-08-03

**Authors:** Wilbert P. Vermeij, Claude Backendorf

**Affiliations:** Laboratory of Molecular Genetics, Faculty of Science, Leiden University, Leiden, The Netherlands; Brunel University, United Kingdom

## Abstract

Wound healing is a complex dynamic process characterised by a uniform flow of events in nearly all types of tissue damage, from a small skin scratch to myocardial infarction. Reactive oxygen species (ROS) are essential during the healing process at multiple stages, ranging from the initial signal that instigates the immune response, to the triggering of intracellular redox-dependent signalling pathways and the defence against invading bacteria. Excessive ROS in the wound milieu nevertheless impedes new tissue formation. Here we identify small proline-rich (SPRR) proteins as essential players in this latter process, as they directly link ROS detoxification with cell migration. A literature-based meta-analysis revealed their up-regulation in various forms of tissue injury, ranging from heart infarction and commensal-induced gut responses to nerve regeneration and burn injury. Apparently, SPRR proteins have a far more widespread role in wound healing and tissue remodelling than their established function in skin cornification. It is inferred that SPRR proteins provide injured tissue with an efficient, finely tuneable antioxidant barrier specifically adapted to the tissue involved and the damage inflicted. Their recognition as novel cell protective proteins combining ROS detoxification with cell migration will provide new venues to study and manage tissue repair and wound healing at a molecular level.

## Introduction

Reactive oxygen species (ROS) are produced directly after tissue damage and are essential during various stages of the healing process, ranging from the initial signal that instigates the immune response to the defence against invading bacteria [1], [2]. For efficient healing, the injured tissue has to rapidly adapt via ROS detoxification to allow correct regulation of redox-sensitive signalling pathways implicated in the healing process [2]–[4]. This rapid response is crucial in all tissues especially during injury, notably in the heart and brain [5]. Due to its accessibility, cutaneous wounding, where a temporal defect results in a local loss of the skin's protective barrier function [6], [7], is particularly well studied. This barrier, which normally provides protection against external insults, is maintained, restored and continuously self-renewed by proliferating, migrating and terminally differentiating keratinocytes [8], [9]. When basal keratinocytes initiate the differentiation process, they undergo morphological changes and express several cornified envelope precursor proteins, such as involucrin, loricrin, and the small proline-rich (SPRR) proteins, ending as a layer of dead flattened cells on the skin surface. In this upper cornified layer, the precursor proteins are cross-linked at the cell periphery and form together with lipids the cornified cell envelope (CE), a structure which is responsible for the major barrier function of the skin [10], [11]. The SPRR gene family is part of the epidermal differentiation complex (EDC) localised on human chromosome 1q21 and mouse chromosome 3. Many of the genes in this locus are co-ordinately regulated during epidermal differentiation. SPRRs are specifically known as stress-inducible proteins involved in the adaptation of the skin barrier following various forms of stress [12]. During wound healing keratinocytes are the first cells to adapt to defects in the barrier and switch from a differentiating to a migratory mode in order to form new tissue [7], [13]. Migrating keratinocytes lack the CE and therefore an alternative protective barrier appears crucial to enter the highly oxidised wound.

Here we show that SPRR proteins protect keratinocytes from excessive ROS by direct quenching via their cysteine residues. This activity is directly related to their ability to promote cell migration. A literature-based meta-analysis disclosed that SPRR proteins do not only exert this function in squamous epithelia but also in a wide range of other major tissues and organs.

## Results and Discussion

**Figure 1:**
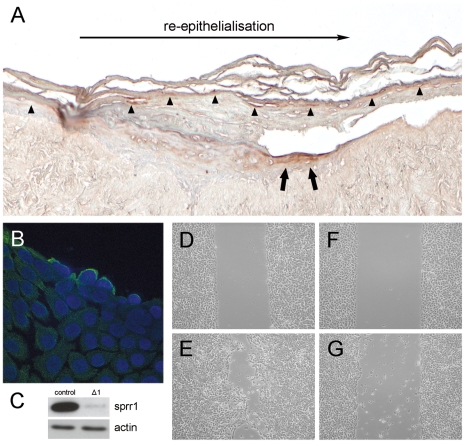
Involvement of SPRR proteins in wound healing and cell migration. A, Immunohistochemistry of SPRR1 expression (brown) in full-thickness wounds obtained after N_2_ freezing [14]. Expression in the migrating front cells at the edge of the wound is indicated by large arrows. Differentiation-mediated expression in the upper layers of the epidermis is indicated by arrow-heads. B, Immunofluorescence detection of SPRR1 expression (green) in cultured OKF keratinocytes 8 h after scratch-wounding. Note the preferential localisation of SPRR1 at the periphery of the cells at the edge of the scratch-wound. C, SPRR Western blot analysis of control OKF and shRNA downregulated OKF-Δ1 cells. Actin expression is used as internal control. D–G, Inhibition of cell migration in SPRR knockdown cells. OKF control (D, E) and OKF-Δ1 knockdown cells (F, G) were compared in a scratch-wound assay. Pictures D, F, were taken immediately after scratch-wounding, whereas pictures E, G, were taken after a subsequent 48 h incubation period.

**Figure 2:**
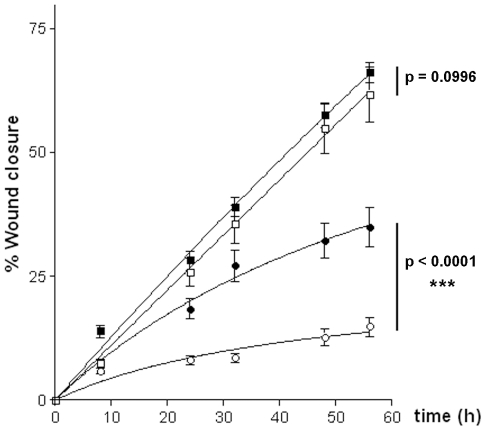
Effect of ascorbate on scratch-wound closure. Quantification of scratch closure at the indicated time-points in confluent OKF control cultures (square symbols) or OKF-Δ1 SPRR-knockdown cells (circle symbols) either treated (solid symbols) or untreated (open symbols) with 10 µM ascorbate following scratch-wounding. Data show mean +/− s.e.m. of at least 6 independent measurements per time-point. Curves from treated and untreated cultures were analysed via two-way ANOVA by using the Graphpad Prism software: p<0.05 values were considered to indicate a significant difference.

While analysing wounded three-dimensional human skin equivalents (HSE) [14] we found that SPRR proteins showed massive expression at the migrating front (Figure 1A; arrows), which exceeds their normal expression in the upper layers of the skin (arrowheads). SPRR proteins were preferentially found in those cells that are in close contact with the wounded site, suggesting a role in cell migration and new tissue formation. This was substantiated by using a scratch wound assay performed on cultured human keratinocytes. Immunofluorescence staining showed, similar to the HSE data, elevated SPRR expression at the edge of the wound, localising preferentially to the cell periphery of the front row of migrating cells (Figure 1B). This pattern is observed along the whole length of the scratch, although in a patchy fashion. Indeed, the most protruding migrating cells show the highest expression of SPRR at the cell periphery (Figure 1B). This localisation at the moving edge is also supported by live-cell-imaging of pEGFP-SPRR1B transfected keratinocytes, further accentuating the presence of SPRR in membrane ruffles at the migrating front of the cell (Movie S1). In order to find evidence for a direct link between SPRR expression and cell migration, we established keratinocyte cell lines expressing shRNA specifically targeting SPRR genes (OKF-Δ1; see Material and methods) or carrying the empty expression vector (OKF). Under normal culture conditions both cell lines were undistinguishable and showed similar population doublings (data not shown), although in the shRNA expressing cells (OKF-Δ1), SPRR expression was clearly down-regulated (Figure 1C). When comparing the ability of wound closure however, a remarkable difference was perceived between the control and shRNA expressing keratinocytes. Whereas control keratinocytes started to migrate after scratch-wounding (compare Figure 1D and 1E), resulting in a substantial closure after 48 hours, this process was significantly retarded in the SPRR down-regulated cells (Figure 1F and 1G). Quantification substantiated that the shRNA expressing cells lack the ability of rapidly closing the wound (Figure 2, compare the 2 lines with open symbols), indicating that SPRR expression after wounding activates the cell migration process.

**Figure 3:**
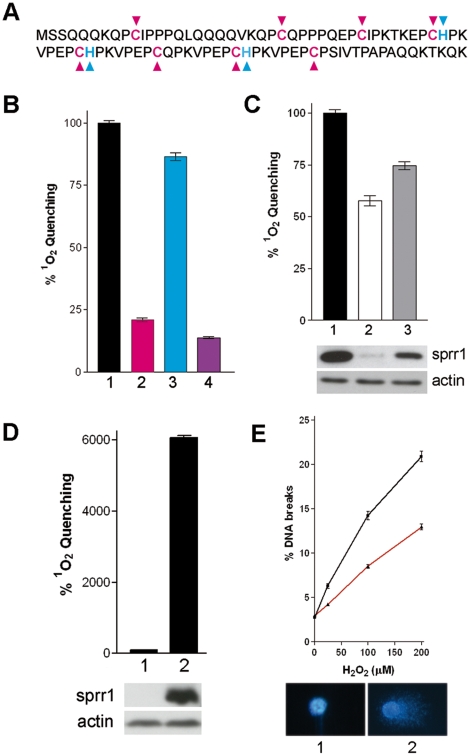
Quenching of Reactive Oxygen Species by SPRR proteins *in vitro* and *in vivo*. A, Representation in one-letter code of the human SPRR1B protein sequence: cysteine (red) and histidine (blue) residues are indicated. B, Relative singlet oxygen quenching potential of equimolar solutions of purified SPRR1B protein either untreated (bar 1, black), NEM-treated (bar 2, red), DEPC-treated (bar 3, blue) or treated with both reagents (bar 4, violet). The quenching ability of untreated protein was set at 100%. C, Quantification of singlet oxygen quenching in living OKF cells expressing various amounts of SPRR proteins. SPRR1 expression levels are indicated below the bar graph (1: control cells; 2: OKF-Δ1; 3: OKF-Δ2). D, Quantification of singlet oxygen quenching in living HeLa cells. Both control (bar 1) and HeLa cells overexpressing SPRR1B [29] (bar 2) are shown. E, Measurement of DNA strand breaks induced in HeLa control cells (black graph) and cells ectopically expressing SPRR1B (red graph) by using the comet assay [20]. For each cell line at least 200 individual cells were quantified. Inserts 1 and 2 represent respectively untreated and H_2_O_2_ treated single HeLa cells stained with DAPI fluorescent DNA stain.

By taking into account the previously postulated role of SPRR proteins as ROS quenchers [15], their localisation in the moving front of migrating cells directly contacting the wounded area (Figure 1) and the importance of ROS regulation and signalling during wound healing [1]–[3], [16], we inferred that the antioxidant properties of SPRR proteins might constitute the key for their implication in cell migration and wound healing. Consequently, it was important to first characterise the ROS quenching abilities of SPRR proteins at a molecular level. Flash-photolysis was used to quantify the life-time of singlet oxygen, a major oxidizing species in skin [17], either in solution in the presence of purified SPRR proteins or directly in living cells. SPRR proteins contain besides proline high amounts of cysteine (5–18%; Figure 3A), known to be involved in ROS quenching [18] and signalling [3], [16]. As expected, SPRR1B efficiently quenched singlet oxygen and this quenching was largely inhibited by specifically targeting cysteine residues with the sulfhydryl reagent N-ethylmaleimide (NEM) (Figure 3B, compare bars 1 & 2). Histidine residues in SPRR1B contributed also to ROS quenching, but to a lesser extent (DEPC treatment, bar 3). The quenching ability of the related SPRR4 protein [19], which contains cysteine but no histidine residues, was not affected by DEPC, while treatment with NEM resulted in a loss of 96% of its quenching activity (see Figure S1). These experiments directly implicate cysteine residues in ROS quenching by SPRR proteins. Flash-photolysis was also used to measure ROS quenching in living cells. OKF-Δ1 and OKF-Δ2 cells, where SPRR expression is downregulated to a different extent (Figure 3C), were compared to OKF control cells carrying the empty expression vector. The data clearly showed that down regulation of SPRR results in a lower quenching potential. Similarly, ectopic expression of SPRR1B in HeLa cells, which do not express SPRR proteins, resulted in a substantial potentiating of ROS quenching (Figure 3D). The fact that these differences could be measured in the context of whole cells proves that SPRR proteins constitute a key determinant for intracellular ROS quenching. The major cellular impact of ROS in living cells is mediated via the induction of DNA strand breaks. In a comet assay [20] HeLa cells, ectopically expressing SPRR1B (Figure 3E, red curve), were significantly more resistant to H_2_O_2_ induced DNA breaks than HeLa control cells (black curve). These experiments suggest a direct and major involvement of SPRR proteins in intracellular ROS detoxification. To assess the importance of ROS detoxification during wound healing, the scratch-wounded OKF-Δ1 cells were treated with ascorbate, a well known ROS quencher. The loss of migratory ability due to SPRR down-regulation (Figure 2, compare open circles and squares) can at least be partially reverted by ascorbate (solid circles). Interestingly, ascorbate did not affect the migratory properties of SPRR expressing control cells (compare open and solid squares). Apparently, the enhancement of cell migration is not a general effect of ascorbate, but it is only apparent in a SPRR-deficient background, proving that SPRR mediated ROS detoxification is directly responsible for efficient cell migration.

**Figure 4:**
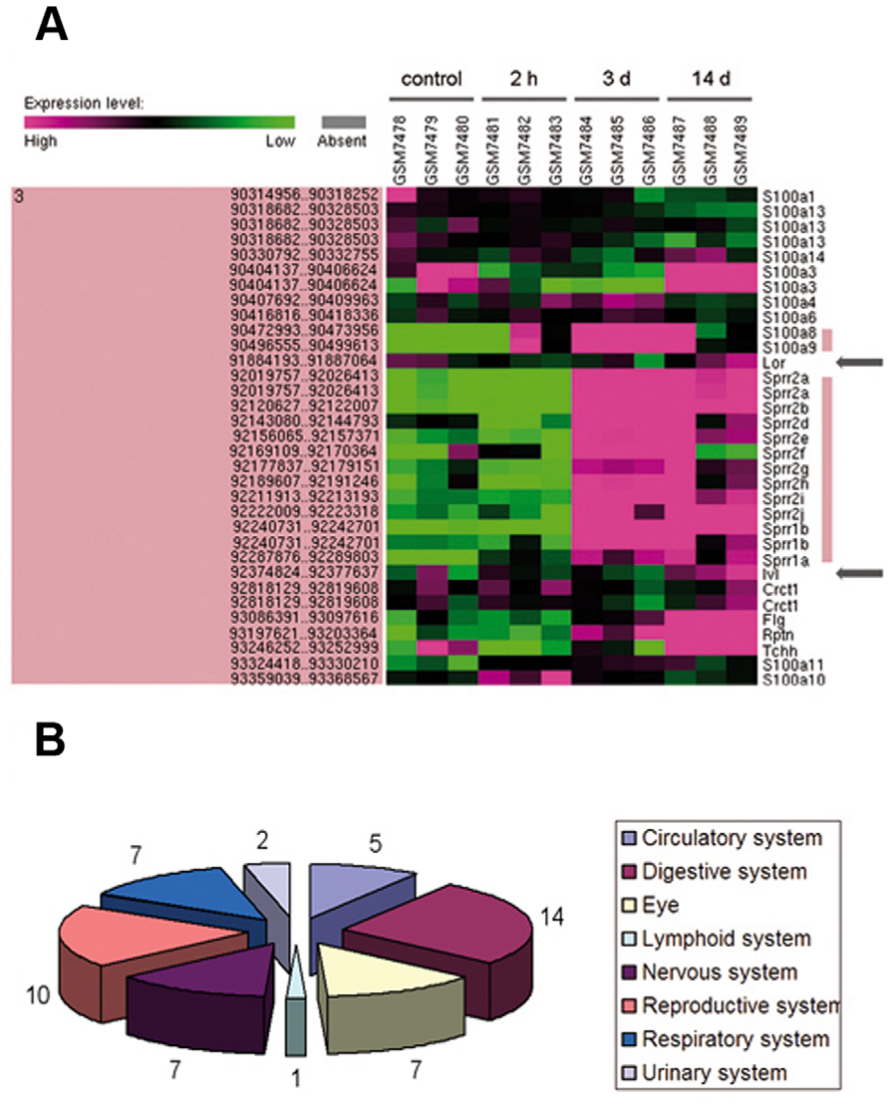
Meta-analysis of SPRR expression during cutaneous wound healing and in non-squamous tissues. A, Effect of cutaneous burn wounding on gene expression in the epidermal differentiation complex (EDC) on mouse chromosome 3. Microarray data are from the Gene Expression Omnibus repository (http://www.ncbi.nlm.nih.gov/geo/ dataset GDS353). Expression levels of 4 experimental conditions are shown: unburned skin (control), burn skin harvested 2 hours (2h), 3 days (3d) and 14 days (14d) after injury [22]. The positions of involucrin and loricrin are indicated by arrows. The SPRR gene family and the S100A8 and S100A9 genes are marked with pink bars. B, Graphical representation of the number of studies describing SPRR up-regulation in non-squamous tissues (grouped per organ) following stress or injury. The corresponding references are supplied in References S1.

In order to further validate these *in vitro* experiments by *in vivo* observations, we performed a meta-analysis on recent genomic/proteomic screens and were able to find evidence for up-regulation of SPRR expression during wound healing in the skin. Cooper and co-workers analysed gene-expression in wounded skin from neonatal mice at different time points by clustering genes with similar expression patterns. Although not specifically mentioned by the authors, the analysis revealed several SPRRs among the most highly up-regulated genes, together with proteins with a known antioxidant function, all involved in tissue repair [21]. Feezor and co-workers have analyzed temporal patterns of global gene expression following infliction of murine cutaneous burn injuries [22]. We compared the expression of EDC genes during wound healing in their dataset available via the Gene Expression Omnibus repository (dataset GDS353; http://www.ncbi.nlm.nih.gov/geo/). SPRR genes (Figure 4A, large pink bar) have a very characteristic expression pattern which is clearly different from the classical cornified envelope precursor proteins involucrin and loricrin (indicated by arrows) and is only matched by S100A8 and S100A9 (short pink bar), two genes previously implicated in wound healing [23].

The above data provide insight into a more global function for SPRR proteins in wound healing that might not be restricted to stratified squamous epithelia. Along these lines of thinking, we extended our literature-based meta-analysis to other tissues. This analysis proved that SPRR genes are highly up-regulated in more than 50 microarray screens from divergent tissues and cell types (Figure 4B), often linked to stress or tissue remodelling. For example, in cardiomyocytes, SPRR was identified as a cardioprotective protein after ischemic stress [5]. During ischemia/reperfusion high levels of ROS are produced in the heart, which can be detoxified by massively increased SPRR expression in the same way as shown here for cultured cells. SPRR protein levels increase by more than 200 fold in response to bacterial colonization of the intestine [24] and here SPRRs are also likely to fulfil an antioxidant function. Bile duct ligation resulted in SPRR expression and subsequent adaptation of the biliary barrier [25]. It is interesting to mention that also in this specific case a direct link has been laid between SPRR expression and *in vitro* cell migration of human biliary epithelial cells [26]. During development of the mammary gland [27] as well as during nerve regeneration after axotomy [28], SPRR proteins appear to function as tissue remodellers. In the injured axons, SPRR expression was mainly found in the axonal growth cones where it localises to cellular membrane ruffles, very similar to the situation described here for migrating keratinocytes. Additionally the same study has revealed that down-regulation of SPRR in axotomized neurons impaired directed axonal outgrowth [28].

The above data disclose a novel and unexpected role of SPRR proteins in global wound healing, which links ROS protection and tissue remodelling. How can this function be reconciled with their canonical function restricted to skin keratinisation? Previously we have proposed that the differential regulation of the 11 members of the SPRR gene-family provides a molecular mechanism for effectively adapting the barrier function in the uppermost layer of our skin. This differential regulation of highly homologous genes with redundant functions that respond selectively to various forms of stress or tissue requirements, allows a tightly regulated protein dosage that provides optimal barrier function to different squamous epithelia, while allowing adaptation to diverse external insults [12]. In the same way, a similar protein dosage mechanism is also likely to provide all tissues with an efficient, finely tuneable antioxidant barrier, specifically adapted to the tissue involved and the damage inflicted. The recognition of SPRRs as efficient cell protective proteins, linking ROS detoxification with cell migration, will provide new possibilities to study and manage tissue repair and wound healing at a molecular level.

## Materials and Methods

### Human skin equivalents

Sections from full-thickness human skin equivalent wounds, induced by liquid nitrogen freezing, were obtained from Dr. A. El Ghalbzouri (Department of Dermatology, Leiden University Medical Centre) and were previously described [14]. Expression of SPRR was assessed by immunostaining using a monospecific rabbit-antibody [29] and counterstained with hematoxylin. The mono-specificity of the antibodies was tested on HeLa cells (which do not express SPRR proteins), transfected with the various SPRR family members, both for Western blotting and immunostaining [29].

### shRNA mediated knockdown

For the downregulation of SPRR, we constructed an episomal expression vector, named pESuper, derived from pECV25 (ATCC) and a fragment of pSuper [30] containing the H1-RNA promoter and polylinker sequence. Oligonucleotides containing the SPRR specific target sequences GCAGTGCAAGCAGCCCTGC (siRNA1) and CGCCAAAGTGCCCAGAGCC (siRNA2) were used for the shRNA-mediated knockdowns. They target both SPRR1 and SPRR2 genes. Immortal OKF keratinocytes (OKF6/TERT-2 [31]), which do not express SPRR3 and SPRR4 (our unpublished observations) were kindly provided by Dr. J.G. Rheinwald (Harvard Medical School, Boston), cultured in defined Keratinocyte-SFM (KSFM; GIBCO) as described [31] and transfected with the above mentioned pESuper constructs, or empty vector control, by using the Amaxa nucleofector according to the manufacturer's protocol (Lonza AG, Cologne, Germany). Stable cell-lines, designated OKF-Δ1 (OKF cells containing the pESuper vector with siRNA1), OKF-Δ2 (containing pESuper with siRNA2), and OKF control cells (empty pESuper vector), were grown until confluence. One day thereafter scratch wounds were made with a yellow pipette tip, the medium was exchanged for KSFM without growth additives and photographs were taken at the indicated time-points. The area remaining free of cells was quantified using Adobe Photoshop and Biorad Quantity One software.

### Protein chemistry

SPRR proteins were produced by using IPTG induction of Escherichia *coli* BL21 (DE3)*RP (Stratagene, La Jolla) bacteria transformed with a pET-vector (Novagen) containing a full-length SPRR cDNA insert. Bacterial pellets were lysed by freeze-thawing in 25mM sodium-citrate (pH 3.6), 1mM EDTA, 1mM DTT. Under these conditions SPRR proteins remained soluble. Upon centrifugation at 37,000 rpm in a Ti60 rotor (Beckman) the supernatant was further purified using a 6 ml Resource S column (GE Healthcare). The buffer was exchanged to 10mM sodium-phosphate (pH 7.0) and the SPRR proteins were stored at −80°C. Sulfhydryl groups of cysteines were modified with N-ethylmaleimide (NEM) and histidine residues with diethylpyrocarbonate (DEPC). The purity of all proteins as well as all modifications were confirmed by mass spectrometry.

### ROS measurements and toxicity assays

Flash-photolysis experiments were performed using a Continuum Surelite I YAG-laser. ^1^O_2_ was detected with a Judson Germanium G-050 photodiode coupled to a Judson preamplifier. All samples were measured in a glass cuvette with magnetic stirrer in D_2_O containing Rose Bengal as sensitizer. The comet assay was performed as previously described [20] and quantified using ColourProc, an in-house software program kindly provided by Dr. H. Vrolijk (Department of Molecular Cell Biology, Leiden University Medical Center).

## Supporting Information

Figure S1 Reactive oxygen quenching of SPRR4 protein *in vitro*. A, Representation in one-letter code of the human SPRR4 protein sequence: cysteine residues are indicated in red. Note that SPRR4 does not contain histidine residues. B, Relative singlet oxygen quenching potential of equimolar solutions of purified SPRR4 protein either untreated (bar 1, black), NEM-treated (bar 2, red), DEPC-treated (bar 3, blue) or treated with both reagents (bar 4, violet). The quenching ability of untreated protein was set at 100%.
(4.63 MB TIF)

References S1 References corresponding to meta-analysis of SPRR expression in non-squamous tissues.
(0.07 MB DOC)

Movie S1 Involvement of SPRR proteins in cell migration. OKF keratinocytes were transiently transfected with pEGFP-SPRR1B and analyzed with the Olympus IX81 live-imaging station. Time-lapse images were recorded for 200 min at intervals of 2 min. Note the localization of SPRR proteins to membrane ruffles at the leading edge of migrating cells. This pattern was not observed in parallel cultures expressing pEGFP-actin or pEGFP-tubulin fusion proteins and was confirmed by SPRR-antibody staining. SPRR proteins were also consistently found in the nuclei of these cells.
(3.12 MB MOV)

## References

[1] Niethammer P, Grabher C, Look AT, Mitchison TJ (2009). A tissue-scale gradient of hydrogen peroxide mediates rapid wound detection in zebrafish.. Nature.

[2] Schafer M, Werner S (2008). Oxidative stress in normal and impaired wound repair.. Pharmacol Res.

[3] Sen CK, Roy S (2008). Redox signals in wound healing.. Biochim Biophys Acta.

[4] Singer AJ, Clark RA (1999). Cutaneous wound healing.. N Engl J Med.

[5] Pradervand S, Yasukawa H, Muller OG, Kjekshus H, Nakamura T (2004). Small proline-rich protein 1A is a gp130 pathway- and stress-inducible cardioprotective protein.. Embo J.

[6] Martin P (1997). Wound healing–aiming for perfect skin regeneration.. Science.

[7] Gurtner GC, Werner S, Barrandon Y, Longaker MT (2008). Wound repair and regeneration.. Nature.

[8] Candi E, Schmidt R, Melino G (2005). The cornified envelope: a model of cell death in the skin.. Nat Rev Mol Cell Biol.

[9] Nemes Z, Steinert PM (1999). Bricks and mortar of the epidermal barrier.. Exp Mol Med.

[10] Kalinin AE, Kajava AV, Steinert PM (2002). Epithelial barrier function: assembly and structural features of the cornified cell envelope.. Bioessays.

[11] Roop D (1995). Defects in the barrier.. Science.

[12] Cabral A, Voskamp P, Cleton-Jansen AM, South A, Nizetic D (2001). Structural organization and regulation of the small proline-rich family of cornified envelope precursors suggest a role in adaptive barrier function.. J Biol Chem.

[13] Freedberg IM, Tomic-Canic M, Komine M, Blumenberg M (2001). Keratins and the keratinocyte activation cycle.. J Invest Dermatol.

[14] El Ghalbzouri A, Hensbergen P, Gibbs S, Kempenaar J, van der Schors R (2004). Fibroblasts facilitate re-epithelialization in wounded human skin equivalents.. Lab Invest.

[15] Alia A, Matysik J, Backendorf CMP (2003). Use of proline and functional equivalents thereof and compositions containing said compounds.. International Patent: WO 03/075903.

[16] D'Autreaux B, Toledano MB (2007). ROS as signalling molecules: mechanisms that generate specificity in ROS homeostasis.. Nat Rev Mol Cell Biol.

[17] Kochevar IE (2004). Singlet oxygen signaling: from intimate to global.. Sci STKE.

[18] Michaeli A, Feitelson J (1994). Reactivity of singlet oxygen toward amino acids and peptides.. Photochem Photobiol.

[19] Cabral A, Sayin A, de Winter S, Fischer DF, Pavel S (2001). SPRR4, a novel cornified envelope precursor: UV-dependent epidermal expression and selective incorporation into fragile envelopes.. J Cell Sci.

[20] Collins AR, Dusinska M, Gedik CM, Stetina R (1996). Oxidative damage to DNA: do we have a reliable biomarker?. Environ Health Perspect.

[21] Cooper L, Johnson C, Burslem F, Martin P (2005). Wound healing and inflammation genes revealed by array analysis of ‘macrophageless’ PU.1 null mice.. Genome Biol.

[22] Feezor RJ, Paddock HN, Baker HV, Varela JC, Barreda J (2004). Temporal patterns of gene expression in murine cutaneous burn wound healing.. Physiol Genomics.

[23] Thorey IS, Roth J, Regenbogen J, Halle JP, Bittner M (2001). The Ca2+-binding proteins S100A8 and S100A9 are encoded by novel injury-regulated genes.. J Biol Chem.

[24] Hooper LV, Wong MH, Thelin A, Hansson L, Falk PG (2001). Molecular analysis of commensal host-microbial relationships in the intestine.. Science.

[25] Nozaki I, Lunz JG, Specht S, Stolz DB, Taguchi K (2005). Small proline-rich proteins 2 are noncoordinately upregulated by IL-6/STAT3 signaling after bile duct ligation.. Lab Invest.

[26] Demetris AJ, Specht S, Nozaki I, Lunz JG, Stolz DB (2008). Small proline-rich proteins (SPRR) function as SH3 domain ligands, increase resistance to injury and are associated with epithelial-mesenchymal transition (EMT) in cholangiocytes.. J Hepatol.

[27] Morris JS, Stein T, Pringle MA, Davies CR, Weber-Hall S (2006). Involvement of axonal guidance proteins and their signaling partners in the developing mouse mammary gland.. J Cell Physiol.

[28] Bonilla IE, Tanabe K, Strittmatter SM (2002). Small proline-rich repeat protein 1A is expressed by axotomized neurons and promotes axonal outgrowth.. J Neurosci.

[29] Hohl D, de Viragh PA, Amiguet-Barras F, Gibbs S, Backendorf C (1995). The small proline-rich proteins constitute a multigene family of differentially regulated cornified cell envelope precursor proteins.. J Invest Dermatol.

[30] Brummelkamp TR, Bernards R, Agami R (2002). A system for stable expression of short interfering RNAs in mammalian cells.. Science.

[31] Dickson MA, Hahn WC, Ino Y, Ronfard V, Wu JY (2000). Human keratinocytes that express hTERT and also bypass a p16(INK4a)-enforced mechanism that limits life span become immortal yet retain normal growth and differentiation characteristics.. Mol Cell Biol.

