# Supplementary figures and images for: Skin Cornification Proteins Provide Global Link between ROS Detoxification and Cell Migration during Wound Healing
Source: PLoS One. 2010 Aug 3;5(8):e11957. doi: 10.1371/journal.pone.0011957 (PMC2914756; doi:10.1371/journal.pone.0011957)

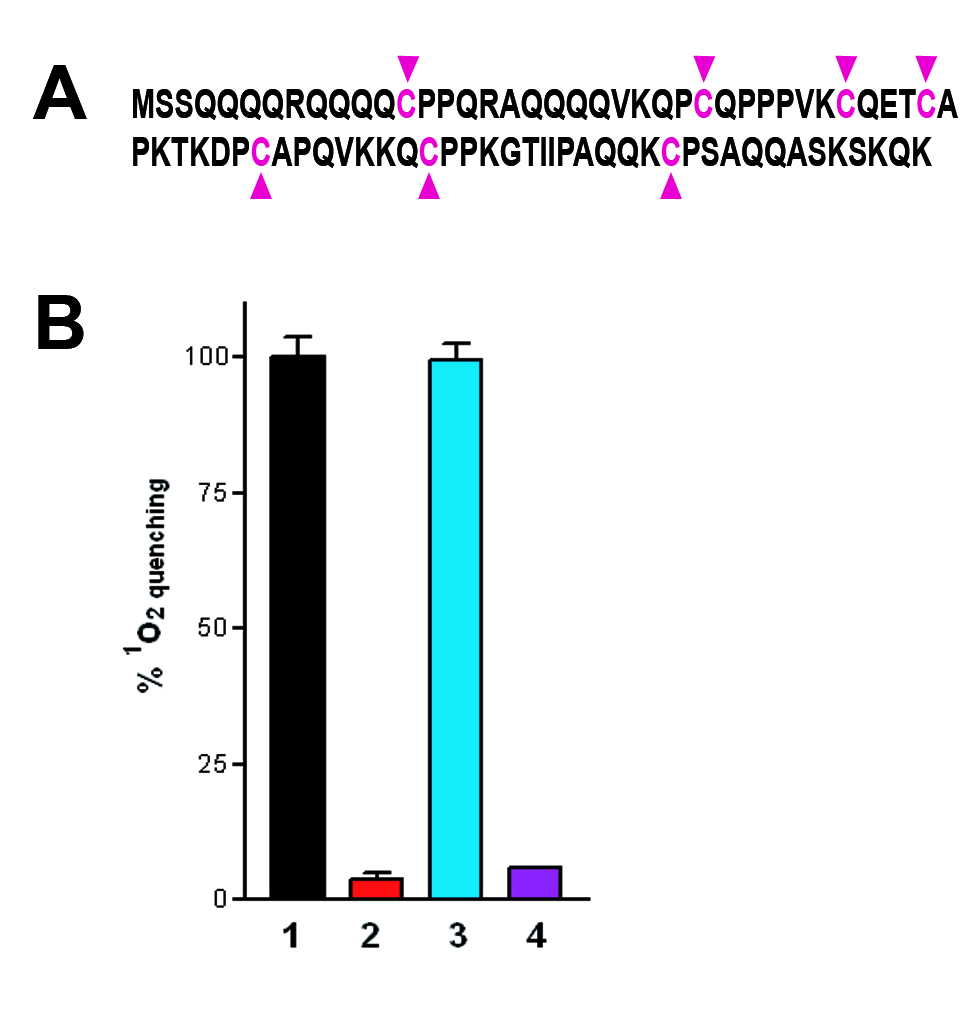

Supplement: Figure S1 — Reactive oxygen quenching of SPRR4 protein in vitro. A, Representation in one-letter code of the human SPRR4 protein sequence: cysteine residues are indicated in red. Note that SPRR4 does not contain histidine residues. B, Relative singlet oxygen quenching potential of equimolar solutions of purified SPRR4 protein either untreated (bar 1, black), NEM-treated (bar 2, red), DEPC-treated (bar 3, blue) or treated with both reagents (bar 4, violet). The quenching ability of untreated protein was set at 100%. (4.63 MB TIF) [file pone.0011957.s001.tif]
