# Supplementary material for: Skin Cornification Proteins Provide Global Link between ROS Detoxification and Cell Migration during Wound Healing
Source: PLoS One. 2010 Aug 3;5(8):e11957. doi: 10.1371/journal.pone.0011957 (PMC2914756; doi:10.1371/journal.pone.0011957)
Supplement: References S1 — References corresponding to meta-analysis of SPRR expression in non-squamous tissues. (0.07 MB DOC) [file pone.0011957.s002.doc]

**Skin cornification proteins provide global link between ROS detoxification and cell migration during wound-healing**

Wilbert P. Vermeij and Claude Backendorf ^*^

*Laboratory of Molecular Genetics, Faculty of Science, Leiden University, P.O. Box 9502, 2300 RA Leiden, The Netherlands*

**Supporting Information:**

**Meta-analysis of SPRR expression in non-squamous tissues.** References corresponding to studies of figure 4B are supplied below and are categorized according to organ type:

**Circulatory system**

1. Ding, J.-H. et al. Dilated cardiomyopathy caused by tissue-specific ablation of SC35 in the heart. *Embo J* 23, 885-896 (2004).
2. Pradervand, S. et al. Small proline-rich protein 1A is a gp130 pathway- and stress-inducible cardioprotective protein. *Embo J* 23, 4517-4525 (2004).
3. Pyle, A.L. et al. Regulation of the atheroma-enriched protein, SPRR3, in vascular smooth muscle cells through cyclic strain is dependent on integrin α1β1/collagen interaction. *Am J Pathol* 173, 1577-1588 (2008).
4. Pyle, A.L. et al. Biomechanical stress induces novel arterial intima-enriched genes: implications for vascular adaptation to stress. *Cardiovasc Pathol* Epub ahead of print doi:10.1016/j.carpath.2008.12.006 (2009)
5. Young, P. P., Modur, V., Teleron, A. A. & Ladenson, J. H. Enrichment of genes in the aortic intima that are associated with stratified epithelium: implications of underlying biomechanical and barrier properties of the arterial intima. *Circulation* 111, 2382-2390 (2005).

**Digestive system**

1. Abgueguen, E. et al. Differential expression of genes related to HFE and iron status in mouse duodenal epithelium. *Mamm Genome* 17, 430-450 (2006).
2. Bracken, S., Byrne, G., Kelly, J., Jackson, J. & Feighery, C. Altered gene expression in highly purified enterocytes from patients with active celiac disease. *BMC Genomics* 9, 377-390 (2008).
3. Demetris, A. J. et al. Wound healing in the biliary tree of liver allografts. *Cell Transplant* 15 Suppl 1, S57-65 (2006).
4. Demetris, A. J., Lunz, J. G., 3rd, Specht, S. & Nozaki, I. Biliary wound healing, ductular reactions, and IL-6/gp130 signaling in the development of liver disease. *World J Gastroenterol* 12, 3512-3522 (2006).
5. Demetris, A.J. et al. Small proline-rich proteins (SPRR) function as SH3 domain ligands, increase resistance to injury and are associated with epithelial-mesenchymal transition (EMT) in cholangiocytes. *J Hepatol* 48, 276-288 (2008).
6. Hooper, L. V. et al. Molecular analysis of commensal host-microbial relationships in the intestine. *Science* 291, 881-884 (2001).
7. Knight, P. A. et al. Expression profiling reveals novel innate and inflammatory responses in the jejunal epithelial compartment during infection with Trichinella spiralis. *Infect Immun* 72, 6076-6086 (2004).
8. Mueller, A. et al. Distinct gene expression profiles characterize the histopathological stages of disease in Helicobacter-induced mucosa-associated lymphoid tissue lymphoma. *Proc Natl Acad Sci U S A* 100, 1292-1297 (2003).
9. Nozaki, I. et al. Small proline-rich proteins 2 are noncoordinately upregulated by IL-6/STAT3 signaling after bile duct ligation. *Lab Invest* 85, 109-123 (2005).
10. Park, Y. K. et al. Gene expression profile analysis of mouse colon embryonic development. *Genesis* 41, 1-12 (2005).
11. Ren, D., Jin, J., Li, X. & Zeng, G. Change of chart genes expression in small intestines of mouse induced by electromagnetic pulse irradiation. *Wei Sheng Yan Jiu* 37, 22-24 (2008)
12. Stern, L. E. et al. cDNA microarray analysis of adapting bowel after intestinal resection. *J Pediatr Surg* 36, 190-195 (2001).
13. Suda, N. Comprehensive gene expression analysis in human periodontal ligaments of the mandibular third molars performing vertical movement and the maxillary second premolars with occlusal contact. *Orthod Craniofac Res* 11, 1-7 (2008).
14. Sun, F. J. et al. Decreased gastric bacterial killing and up-regulation of protective genes in small intestine in gastrin-deficient mouse. *Dig Dis Sci* 48, 976-985 (2003).

**Eye**

1. Chen, Y.-T. et al. Immune profile of squamous metaplasia development in autoimmune regulator-deficient dry eye. *Mol Vis* 15, 563-576 (2009)
2. Chen, Z. et al. Hyperosmolarity-induced cornification of human corneal epithelial cells is regulated by JNK MAPK. *Invest Ophthalmol Vis Sci* 49, 539-549 (2008).
3. De Paiva, C. S. et al. Apical corneal barrier disruption in experimental murine dry eye is abrogated by methylprednisolone and doxycycline. *Invest Ophthalmol Vis Sci* 47, 2847-2856 (2006).
4. De Paiva, C. S. et al. Dry eye-induced conjunctival epithelial squamous metaplasia is modulated by interferon-gamma. *Invest Ophthalmol Vis Sci* 48, 2553-2560 (2007).
5. Kawasaki, S. et al. Up-regulated gene expression in the conjunctival epithelium of patients with Sjogren's syndrome. *Exp Eye Res* 77, 17-26 (2003).
6. Li, S. et al. Small proline-rich protein 1B (SPRR1B) is a biomarker for squamous metaplasia in dry eye disease. *Invest Ophthalmol Vis Sci* 49, 34-41 (2008).
7. Tong, L. et al. Distinct gene subsets in pterygia formation and recurrence: dissecting complex biological phenomenon using genome wide expression data. *BMC Med Genom* 2, 14-34 (2009)

**Lymphoid system**

1. Gotter, J., Brors, B., Hergenhahn, M. & Kyewski, B. Medullary epithelial cells of the human thymus express a highly diverse selection of tissue-specific genes colocalized in chromosomal clusters. *J Exp Med* 199, 155-166 (2004).

**Nervous system**

1. Bonilla, I. E., Tanabe, K. & Strittmatter, S. M. Small proline-rich repeat protein 1A is expressed by axotomized neurons and promotes axonal outgrowth. *J Neurosci* 22, 1303-1315 (2002).
2. Carmichael, S. T. et al. Growth-associated gene expression after stroke: evidence for a growth-promoting region in peri-infarct cortex. *Exp Neurol* 193, 291-311 (2005).
3. Fischer, D., Petkova, V., Thanos, S. & Benowitz, L. I. Switching mature retinal ganglion cells to a robust growth state in vivo: gene expression and synergy with RhoA inactivation. *J Neurosci* 24, 8726-8740 (2004).
4. Li, S. & Strittmatter, S. M. Delayed systemic Nogo-66 receptor antagonist promotes recovery from spinal cord injury. *J Neurosci* 23, 4219-4227 (2003).
5. Lobsiger, C. S., Boillee, S. & Cleveland, D. W. Toxicity from different SOD1 mutants dysregulates the complement system and the neuronal regenerative response in ALS motor neurons. *Proc Natl Acad Sci U S A* 104, 7319-7326 (2007).
6. Marklund, N. et al. Selective temporal and regional alterations of Nogo-A and small proline-rich repeat protein 1A (SPRR1A) but not Nogo-66 receptor (NgR) occur following traumatic brain injury in the rat. *Exp Neurol* 197, 70-83 (2006).
7. Starkey, M.L. et al. Expression of the regeneration-associated protein SPRR1A in primary sensory neurons and spinal cord of the adult mouse following peripheral and central injury. *J Comp Neurol* 513, 51-68 (2009).

**Reproductive system**

1. Hong, S. H. et al. Analysis of estrogen-regulated genes in mouse uterus using cDNA microarray and laser capture microdissection. *J Endocrinol* 181, 157-167 (2004).
2. Hong, S. H. et al. Estrogen regulates the expression of the small proline-rich 2 gene family in the mouse uterus. *Mol Cells* 17, 477-484 (2004).
3. Kouros-Mehr, H. & Werb Z. Candidate regulators of mammary branching morphogenesis identified by genome-wide transcript analysis. *Dev Dyn* 235, 3404-3412 (2006).
4. Mercier, I. et al. Genetic ablation of caveolin-1 drives estrogen-hypersensitivity and the development of DCIS-like mammary lesions. *Am J Pathol* 174, 1172-1190 (2009).
5. Moggs, J. G. et al. Phenotypic anchoring of gene expression changes during estrogen-induced uterine growth. *Environ Health Perspect* 112, 1589-1606 (2004).
6. Morris, J. S. et al. Involvement of axonal guidance proteins and their signaling partners in the developing mouse mammary gland. *J Cell Physiol* 206, 16-24 (2006).
7. Robertson, F. G. et al. Prostate development and carcinogenesis in prolactin receptor knockout mice. *Endocrinology* 144, 3196-3205 (2003).
8. Tan, Y. F., Li, F. X., Piao, Y. S., Sun, X. Y. & Wang, Y. L. Global gene profiling analysis of mouse uterus during the oestrous cycle. *Reproduction* 126, 171-182 (2003).
9. Tan, Y.-F. et al. Gene expression pattern and hormonal regulation of Small Proline-Rich Protein 2 family members in the female mouse reproductive system during the estrous cycle and pregnancy. *Reprod Nutr Dev* 46, 641-655 (2006).
10. Tesfaigzi, J. & Carlson, D. M. Cell cycle-specific expression of G(0)SPR1 in Chinese hamster ovary cells. *Exp Cell Res* 228, 277-282 (1996).

**Respiratory system**

1. Domachowske, J. B., Bonville, C. A., Easton, A. J. & Rosenberg, H. F. Differential expression of proinflammatory cytokine genes in vivo in response to pathogenic and nonpathogenic pneumovirus infections. *J Infect Dis* 186, 8-14 (2002).
2. Rouse, R. L., Boudreaux, M. J. & Penn, A. L. In utero environmental tobacco smoke exposure alters gene expression in lungs of adult BALB/c mice. *Environ Health Perspect* 115, 1757-1766 (2007).
3. Sandler, N. G., Mentink-Kane, M. M., Cheever, A. W. & Wynn, T. A. Global gene expression profiles during acute pathogen-induced pulmonary inflammation reveal divergent roles for Th1 and Th2 responses in tissue repair. *J Immunol* 171, 3655-3667 (2003).
4. Vos, J. B. et al. A molecular signature of epithelial host defense: comparative gene expression analysis of cultured bronchial epithelial cells and keratinocytes. *BMC Genomics* 7, 9-18 (2006).
5. Yoneda, K., Chang, M. M., Chmiel, K., Chen, Y. & Wu, R. Application of high-density DNA microarray to study smoke- and hydrogen peroxide-induced injury and repair in human bronchial epithelial cells. *J Am Soc Nephrol* 14, S284-289 (2003).
6. Zheng, L. et al. Carbon monoxide modulates α-smooth muscle actin and small proline rich-1a expression in fibrosis. *Am J Respir Cell Mol Biol* 41, 85-92 (2009).
7. Zimmermann, N. et al. Expression and regulation of small proline-rich protein 2 in allergic inflammation. *Am J Respir Cell Mol Biol* 32, 428-435 (2005).

**Urinary system**

1. Chen, J. et al. 2,8-Dihydroxyadenine nephrolithiasis induces developmental stage-specific alterations in gene expression in mouse kidney. *Urology* Epub ahead of print doi:10.1016/j.urology.2009.10.031 (2009)
2. Saban, M. R. et al. Repeated BCG treatment of mouse bladder selectively stimulates small GTPases and HLA antigens and inhibits single-spanning uroplakins. *BMC Cancer* 7, 204-218 (2007).
